# Seroprevalence and placental transfer of SARS-CoV-2 antibodies in unvaccinated pregnant women

**DOI:** 10.1186/s12879-024-09399-6

**Published:** 2024-05-21

**Authors:** An Vercoutere, Mbiton Joel Zina, Meltem Telis, Jean-Christophe Goffard, Michel Boulvain, Loïc de Doncker, Sara Derisbourg, Sylvie Houben, Marie-Luce Delforge, Caroline Daelemans, Dorottya Kelen

**Affiliations:** 1 Department of Obstetrics and Gynaecology, Hôpital Erasme, Hôpital Universitaire de Bruxelles (H.U.B.), Route de Lennik 808, 1070 Brussels, Belgium; 2 Outbreak Support Team Carolo, Charleroi Hainaut, Belgium; 3 Department of Neonatology, Hôpital Erasme, Hôpital Universitaire de Bruxelles (H.U.B.), Brussels, Belgium; 4 Department of Internal Medicine, Hôpital Erasme, Hôpital Universitaire de Bruxelles (H.U.B.), Brussels, Belgium; 5 Institute for Medical Immunology, Université Libre de Bruxelles (ULB), Brussels, Belgium; 6 Department of Obstetrics and Gynaecology, Hôpital Delta, Chirec Hospitals, Brussels, Belgium; 7 National Reference Center for Congenital Infections, Hôpital Erasme, Hôpital Universitaire de Bruxelles (H.U.B.), Brussels, Belgium; 8 Department of Woman, Child and Adolescent Medicine, Faculty of Medicine, Geneva University Hospitals, Geneva, Switzerland

**Keywords:** SARS-COV-2 antibodies, Pregnancy, Seroprevalence, Placental transfer, COVID-19, Antibody

## Abstract

**Purpose:**

Pregnant women are at risk of severe SARS-CoV-2 infection, potentially leading to obstetric and neonatal complications. Placental transfer of antibodies directed to SARS-CoV-2 may be protective against neonatal COVID-19, but this remains to be studied. We aimed to determine the seroprevalence of SARS-CoV-2 antibodies in a population of unvaccinated pregnant women and to determine the placental transfer of these antibodies.

**Methodology:**

A total of 1197 unvaccinated women with mostly unknown pre-study SARS-CoV-2 infection status, were tested at delivery for SARS-CoV-2 spike protein IgG antibodies during the first year of the pandemic. Umbilical cord samples were collected and assessed for seropositivity if the mother was seropositive. Maternal characteristics, pregnancy and neonatal outcomes and data on SARS-CoV-2 infection were extracted from medical records.

**Results:**

Specific IgG were detected in 258 women (21.6%). A significant placental transfer to the newborn was observed in 81.3% of cases. The earlier in the 2nd and 3rd trimesters that the mother had contracted the disease and the more symptomatic she was, the greater the likelihood of transplacental transfer of IgG to her newborn.

**Conclusion:**

Approximately one in five women had detectable anti-SARS-CoV-2 spike protein IgG antibodies at delivery during the first year of the pandemic, and these antibodies were significantly transferred to their fetuses. This research provides further evidence to better understand the dynamics of the placental transfer of SARS-CoV-2 IgG antibodies from mothers to their newborns, which is necessary to improve vaccination strategies.

**Supplementary Information:**

The online version contains supplementary material available at 10.1186/s12879-024-09399-6.

## Highlights

- In our large cohort of unvaccinated women, almost ten times the size of the largest previously published study, one in five women had detectable anti-SARS-CoV-2 spike protein IgG antibodies at delivery during the first year of the pandemic.

- Four out of five women transferred these anti-SARS-CoV-2 spike protein IgG antibodies towards their fetuses.

- The earlier the mother was infected—in the 2nd and 3rd trimester—the higher her antibody level, and the more symptomatic she was, the greater the likelihood of transplacental transfer of IgG to her newborn.

- As placental transfer is variable and depends on several factors, understanding the dynamics of antibody transfer and its impact on fetal health following a natural SARS-CoV-2 infection, can improve vaccination strategies to optimize neonatal protection.

**Supplementary Information:**

The online version contains supplementary material available at 10.1186/s12879-024-09399-6.

## Background

The severe acute respiratory syndrome coronavirus (SARS-CoV-2) has spread rapidly around the world, infecting millions of people. Although pregnant women are not more likely to contract the disease, they are more susceptible to develop a severe infection and maternal and pregnancy complications, depending on the variant [1–7].

Several studies have explored the placental transfer of maternal SARS-CoV-2 specific antibodies following maternal infection, the largest cohort published to date – to our knowledge – evaluated 145 mothers [8–16]. There remains a need for more extensive studies to improve our understanding of the complex dynamics of placental transfer of IgG following natural SARS-CoV-2 infection, which may pave the way for advances in vaccination campaigns [8–12]. Vertical transmission of the virus is rare and usually associated with a favorable neonatal outcome [1, 17].

The majority of SARS-Cov-2 infected patients produce immunoglobin M (IgM), A (IgA) and G (IgG) antibodies against the viral spike (S) and nucleocapsid (N) proteins. Detectable IgM appear 6 to 14 days after the onset of symptoms, while IgG become detectable one to three weeks later. IgG reach high titers, decline within two months and then remain relatively stable for the next 6 to 12 months [18, 19].

The fetus produces IgG and IgM antibodies from approximately 20 weeks of gestation. Maternal IgG antibodies are transferred across the placenta to the fetus from the end of the first trimester of pregnancy onwards [20–23], so most of the fetal IgG antibodies are of maternal origin [24]. Gestational age (GA), IgG subclass (highest for IgG1 and lowest for IgG2), antigen specificity, Fc IgG glycosylation, maternal antibody concentration, chronic maternal infection, placental pathology are factors that can influence placental transfer [21, 23, 25–27]. IgM antibodies, on the other hand, do not cross the placenta. If present in the fetal blood, they are deemed to have been produced by the fetus in response to an in utero exposure to SARS-CoV-2, strongly suggesting intrauterine infection [9, 24, 28]. However, cord blood IgM assays are prone to false-positive results due to cross-reactivity or to interference caused by sample contamination with maternal blood or increased permeability of the syncytiotrophoblast barrier due to infection-induced inflammation [10, 29].

### Objectives

The first objective of our study was to report the seroprevalence of SARS-CoV-2 specific IgG antibodies against S protein in unvaccinated pregnant women during the first year of the pandemic. The second objective was to assess the placental transfer of IgG S SARS-CoV-2 antibodies and their determinants and the prevalence of IgM antibodies in the umbilical cord (UC).

### Study design

This prospective, multicenter observational study was conducted between August 18, 2020, and April 2, 2021.

Pregnant women who were admitted for delivery after 35 weeks gestation to Erasme University Hospital (an academic tertiary hospital with about 2000 deliveries per year) or Delta Hospital (a general secondary hospital with about 3500 deliveries per year) in Brussels (Belgium) during the study period were invited to participate. A total of 1207 pregnant women and their newborns were enrolled in the study. Ten cases were excluded from the analysis for the following reasons: duplication (eight cases); no laboratory sample received (one case); incorrect labelling of blood samples (one case).

At the time of enrollment, we obtained informed consent from the participating women and collected blood samples for the measurement of SARS-CoV-2 spike-specific antibodies. In addition, after delivery, whether by delayed or immediate umbilical cord clamping, we collected cord blood samples for the assessment of SARS-CoV-2 specific antibodies. Both blood samples were stored in the BioBank of the National Reference Centre for Congenital Infections and form the Covid VERtical Transmission (CoVerT) cohort.

Detailed information was extracted from the medical records using a standardized data collection form. Data were recorded anonymously in a REDCap database (Research Electronic Data Capture) [30]. For each included case, maternal characteristics (sociodemographic characteristics, past medical history, height, weight, pregnancy complications, obstetric outcome), information on maternal SARS-CoV-2 infection (nasopharyngeal polymerase chain reaction test (PCR), symptoms, hospitalization, imaging, intensive care unit (ICU) admission, oxygen therapy) and neonatal characteristics (birth parameters, neonatal outcome, admission to the neonatal ICU mode of feeding, length of hospital stay) were recorded.

Serological testing was performed using the DiaSorin test on the LIAISON XL analyzer. These tests are based on the detection of antibodies against the spike protein (S), anchored in the viral envelope. Specific IgG were measured against both subunits of the spike protein S1 and S2. These proteins are, respectively, responsible for the binding (S1) and fusion (S2) of the virus to the cell. Only IgM antibodies directed against S1 were evaluated. The diagnostic sensitivity and specificity of the LIAISON SARS-CoV-2 IgM assay were 98.3% (95% CI: 93.9%—99.5%) and 99.2% (95% CI: 98.0%—99.7%), respectively [31]. The IgM cut-off was set at 1.10 AU/mL according to the manufacturer's recommendations. A performance study of the LIAISON SARS-CoV-2 S1/S2 IgG test indicated that the cut-off for IgG positivity was 6.1 AU/mL [31], and we used this cut-off for our study. The specificity and sensitivity using this threshold were 99% (95% CI: 93.0%—100.0%) and 100% (95% CI: 92.0%—100.0%), respectively [31]. All maternal blood samples were tested for IgG. UC samples were tested for IgG and IgM if the maternal result was positive.

Grand multiparity was defined as a participant having given birth three times or more. A seropositive mother is one who tested positive for specific SARS-CoV-2 IgG antibodies in her blood. In our study, a seropositive newborn is defined as an infant whose umbilical cord blood tested positive for specific SARS-CoV-2 IgG antibodies.

The placental transfer ratio (PTR) was calculated as the level of IgG antibodies in UC blood divided by the level of maternal IgG antibodies.

Statistical analyses were performed using STATA version 17 (Statacorp, TX, USA). Descriptive analyses were performed with presentation of numbers and proportions (categorical variables); for quantitative variables mean and standard deviation (in case of a normal distribution), median with interquartile range (in case of an abnormal distribution of the variables) were calculated. For categorical variables, bivariable comparisons were made using the Chi^2^ or Fisher’s exact tests, and for continuous variables, a t-test (two groups) in case of a normal distribution of variables and a Mann–Whitney test (two groups) in case of an abnormal distribution of variables. Multivariable modeling was performed to predict maternal seroprevalence and to identify factors independently associated with the placental transfer. Factors were included in the model if there was an association with a p-value < 0.2 in the univariate analysis. Statistical significance was defined as a p-value < 0.05 and we report the 95% confidence interval (CI).

The study was approved by the central ethics committee of the Erasme University Hospital (ULB) (P2020/396) and by the local ethics committee of the Delta Hospital.

## Results

### Study population

A total of 1197 unvaccinated pregnant women were included in the study. Sociodemographic characteristics are shown in Table 1. The prevalence of positive maternal IgG was higher in women from sub-Saharan Africa (odds ratio OR 2.69) and North Africa (OR 2.16), compared to women from Northern Europe (*p* = 0.001). IgG-positive participants were more likely to be large multiparas (OR 2.77 compared to nulliparas). No other sociodemographic differences were observed between participants who were positive or negative for IgG against the SARS-CoV-2 spike protein.

**Table 1:** Sociodemographic characteristics of the participants. Comparison between mothers with and without anti-SARS-CoV-2 spike protein IgG antibodies detected

Characteristics	ALL n (%)	Maternal IgG positive	Maternal IgG negative	*p* value (OR)
**Maternal age**	1197	258	939	0.1^*^
18–24	46 (3.8)	14 (5.4)	32 (3.4)	
25–29	275 (23.0)	62 (24.0)	213 (22.7)	
30–34	517 (43.2)	116 (45.0)	401 (42.7)	
35–39	275 (23.0)	45 (17.5)	230 (24.5)	
40 or more	84 (7.0)	21 (8.1)	63 (6.7)	
**Smoking** (14 missing data)				
Yes	50 (4.2)	11 (4.3)	39 (4.2)	1.0^*^
**Drug abuse** (13 missing data)				
Yes	4 (0.3)	0	4 (0.4)	0.6^*^
**Origin**				0.001^*^
Northern Europe	799 (66.8)	152 (58.9)	647 (68.9)	ref
Mediterranean region	182 (15.2)	36 (14.0)	146 (15.6)	OR 1.05 CI [0.70–1.57]
Northern Africa	104 (8.7)	35 (13.6)	69 (7.4)	**OR 2.16 CI [1.39–3.36]**
Sub-Saharan Africa	62 (5.2)	24 (9.3)	38 (4.0)	**OR 2.69 CI [1.56–4.62]**
Other	50 (4.2)	11 (4.2)	39 (4.1)	OR 1.20 CI [0.60–2.40]
**BMI** (39 missing data)				
Underweight (< 18.5)	54 (5.1)	11 (4.9)	43 (5.2)	0.5^*^
Normal weight (18.5 – 25)	719 (68.0)	150 (66.7)	569 (68.3)	
Overweight (25 – 30)	187 (17.7)	37 (16.4)	150 (18.0)	
Obese (≥ 30)	98 (9.3)	27 (12.0)	71 (8.5)	
**Medical history**				
Hypertension	9 (0.8)	3 (1.2)	6 (0.6)	0.4
Pre-existing diabetes	6 (0.5)	1 (0.4)	5 (0.5)	1.0
Asthma	56 (4.7)	12 (4.7)	44 (4.7)	1.0
**Poor obstetric history**				
Late miscarriage	8/1193 (0.7)	2/258 (0.8)	6/935 (0.6)	0.7^*^
Stillbirth	9/1187 (0.8)	2/255 (0.8)	7/932 (0.8)	1.0^*^
Preterm birth < 32 weeks	6/1186 (0.5)	2/256 (0.8)	4/930 (0.4)	0.6^*^
Preterm birth < 37 weeks	23/1192 (1.9)	5/255 (2.0)	18/937 (1.9)	1.0^*^
**Parity**				0.004^*^
Nulliparity	621 (51.9)	122 (47.3)	499 (53.1)	ref
Multiparity 1–2	524 (43.8)	115 (44.6)	409 (43.6)	**OR 1.1 CI [0.86–1.53]**
Grand multiparity (> = 3)	52 (4.3)	21 (8.1)	31 (3.3)	**OR 2.77 CI [1.54–4.99]**

*Abbreviations*: *BMI* Body mass index, *CI* Confidence interval, *IgG* Immunoglobulin of type G, *OR* Odds ratio, *ref* Reference

^*^Fischer’s exact test

Pregnancy characteristics and obstetric outcomes are shown in Table 2. The median GA at delivery was 39 weeks, ranging from 35 weeks 0 days to 42 weeks 2 days. No maternal death was reported. One neonate died from a congenital heart defect (Table 3).

**Table 2:** Pregnancy and obstetric outcomes. Comparison between participants with and without maternal anti- SARS-CoV-2 spike protein IgG antibodies

**Characteristics**	**ALL n (%)**	**Maternal IgG positive** (*n* = 258)	**Maternal IgG negative** (*n* = 939)	*p* value **OR**
**Pregnancy complications**				
None	641 (53.6)	122 (47.3)	519 (55.3)	0.02^*^
Risk premature birth	38 (3.2)	11 (4.3)	27 (2.9)	0.3^*^
PPROM	11 (0.9)	1 (0.4)	10 (1.1)	0.9^^^
Gestational diabetes with diet	94 (7.9)	20 (7.8)	74 (7.9)	0.9^*^
Gestational diabetes with insulin	49 (4.1)	10 (3.9)	39 (4.2)	0.8^*^
Gestational hypertension	17 (1.4)	7 (2.7)	10 (1.1)	0.07^^^
Preeclampsia / HELLP / Eclampsia	25 (2.1)	6 (2.3)	19 (2.0)	0.8^*^
Thrombocytopenia	14 (1.2)	2 (0.8)	12 (1.3)	0.7^^^
Postpartum hemorrhage	17 (1.4)	7 (2.7)	10 (1.1)	0.4^*^
**Cumulative pregnancy complications**				**0.004**^**^**^
0	654 (54.6)	124 (48.1)	530 (56.4)	ref
1	419 (35.0)	95 (36.8)	324 (34.5)	**OR 1.25** CI[0.93–1.69]
2	103 (8.6)	29 (11.2)	74 (7.9)	**OR 1.67** CI[1.04–2.68]
> 2	21 (1.8)	10 (3.9)	11 (1.2)	**OR 3.88** CI[1.61–9.35]
**Type of birth** (3 missing data)				
Vaginal birth	924 (77.4)	206 (80.5)	718 (76.6)	0.6^^^
Instrumental birth	166 (13.9)	33 (12.9)	133 (14.2)	
Primary C-section	18 (1.5)	3 (1.2)	15 (1.6)	
Secondary C-section	86 (7.2)	14 (5.5)	72 (7.7)	
**Gestational age at birth** (4 missing data)				
< 37 weeks	19 (1.6)	2 (0.8)	17 (1.8)	0.3^^^
37 < 38 weeks	71 (6.0)	19 (7.4)	52 (5.6)	
38 < 39 weeks	192 (16.1)	41 (16.0)	151 (16.1)	
39 < 40 weeks	323 (27.1)	80 (31.3)	243 (25.9)	
40 < 41 weeks	402 (33.7)	80 (31.3)	322 (34.3)	
> 41 weeks	186 (15.5)	34 (13.3)	152 (16.2)	

Cumulative pregnancy complications: sum of the pregnancy complications

*Abbreviations*: *CI* Confidence interval, *C-section* Caesarean section, *HELLP* Hemolysis elevated liver enzymes low platelets, *IgG* Immunoglobulins G, *OR* Odds ratio, *pPROM* Preterm premature rupture of membranes, *ref* Reference

^*^Chi2 test

^Fisher’s exact test

**Table 3:** Neonatal outcomes. Comparison between participants with and without maternal anti- SARS-CoV-2 spike protein IgG antibodies

**Characteristics**	**ALL n (%)**	**Maternal IgG positive** (*n* = 258)	**Maternal IgG negative** (*n* = 939)	***p* value**
**Apgar 5 min < 7**	14 (1.2)	3 (1.2)	11 (1.2)	1.0^^^
**pH < = 7.0**	7 (0.6)	0	7 (0.8)	0.5^^^
**NICU admission**	59 (4.9)	17 (6.6)	42 (4.5)	0.2^°^
Length neonatal stay (median, (IQR)	(3.0 (1.0))	(3.0 (1.0))	(3.0 (1.0))	
**Breastfeeding**				
Yes, exclusively	975 (82.6)	213 (83.2)	762 (82.4)	0.9^*^
Yes, but mixed	92 (7.8)	18 (7.0)	74 (8.0)	
No, artificial feeding	114 (9.7)	89 (9.6)	89 (9.6)	
**Birthweight** (gram)				
Mean (se)	3422.7 (12.5)	3435.9 (14.0)	3374.6 (27.1)	0.04^#^
**Neonatal death**	1 (0.08)	1 (0.1)	0	1.0 ^

*Abbreviations*: *IQR* inter quartile range, *se* standard error

^*^Chi2 test

^^^Fisher’s exact test

^°^Mann–Whitney test

^#^Student t-test

### Prevalence of maternal IgG antibodies

**Fig. 1:**
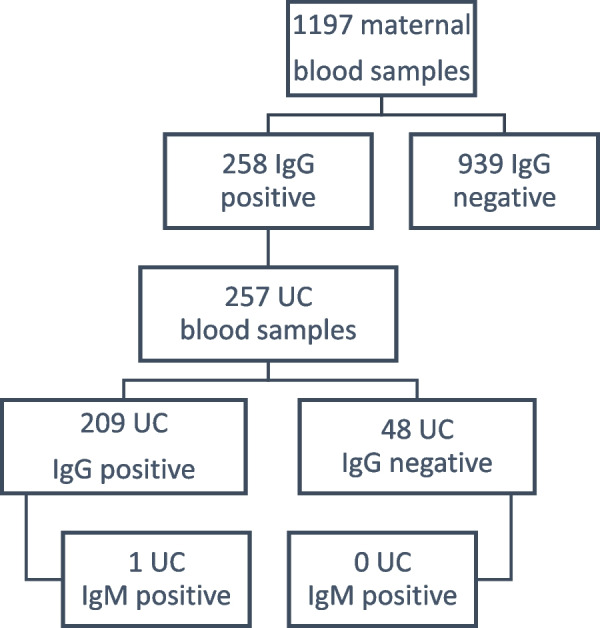
Flowchart representing detected antibodies in blood samples of participants. Abbreviations: IgM = immunoglobulin M; IgG = immunoglobulin G; UC = umbilical cord

A total of 258 women out of 1197 (21.6%) had SARS-CoV-2 S protein spike antibodies when they were admitted to the labor ward (Fig. 1).

We have observed no difference between seropositive and seronegative mothers in terms of mode of delivery or GA at birth. Seropositive mothers were more likely to have pregnancy complications (OR 1.38) and cumulative pregnancy complications (*p* = 0.004) (Table 2). Neonatal outcomes were similar between the two groups (Table 3). We observed a statistically significant difference in birth weight between the two groups; neonates born from seropositive mothers had a lower birth weight (mean 3375 g) compared to the ones born from seronegative mothers (mean 3436 g) (*p* = 0.04), even after adjustment for confounders.

A total of 48.7% (503/1197) participants underwent a PCR testing, mostly on admission or the day before. Among seronegative women, 3.1% of women tested positive compared with 40.4% among seropositive women (*p* < 0.001) (Table S1).

Ninety newborns (7.6%) were tested by nasopharyngeal SARS-CoV-2 PCR in the immediate postpartum period guided by local policies and clinical discretion; 38 (42.2%) were positive, all of these infants were born to IgG-positive mothers.

After adjustment, maternal seropositivity was associated with North African origin (aOR 2.2), large multiparity (aOR 2.8) and history of a positive PCR test for SARS-CoV-2 (aOR 28.7). When no SARS-CoV-2 PCR test was performed, maternal age also influenced seropositivity. Each additional year of maternal age, reduced the risk of seropositivity by 0.05% (Table S2).

### Placental transfer of IgG antibodies

In 209 out of 257 umbilical cord samples collected from seropositive women tested at birth, we detected the presence of IgG antibodies (81.3%) (Fig. 1). When we compared the two subgroups (positive or negative IgG in the UC), no statistically significant differences were found in sociodemographic and maternal characteristics or obstetric outcomes (Table S3-4).

We observed a statistically significant difference in maternal IgG levels between the group of women who had transferred their antibodies to their newborns and those who did not (median IgG level 20.5 AU/ml versus 7.3 AU/ml) (OR 1.2; *p* < 0.001) (Table 4). Mothers with maternal symptoms suggestive of SARS-CoV-2 infection were also more likely to have transferred their IgG (OR 3.8; *p* = 0.04) compared to asymptomatic mothers.

**Table 4:** Association between placental transfer of IgG and variables related to the SARS-CoV2 infection in IgG positive mothers

Umbilical cord samples from women seropositive for SARS-CoV-2					
**Variables related to SARS-CoV-2 infection**	**UC** **IgG positive (*n* = 209; 81.3%)**		**UC** **IgG negative (*n* = 48; 18.7%)**		***P* value** **OR**
	**n (%)**	**Median (IQR)**	**n (%)**	**Median (IQR)**	
**Median maternal IgG level** (U/mL)		20.5 (11.6–37.2)		7.3 (6.8–9.4)	** < 0.001ͣ** **OR 1.2**
**Symptoms**					
Yes	38 (18.2)		3 (6.3)		**0.04**^b^
No	171 (81.8)		45 (93.7)		**OR 3.3**
**Number of symptoms**					
0	171 (81.8)		45 (93.7)		0.37^d^
1	17 (7.7)		2 (4.2)		
2	5 (2.4)		1 (2.1)		
3	6 (2.9)		0		
4 or more	10 (4.8)		0		
Maternal nasopharyngeal PCR^*^	*n* = 148		*n* = 17		1.0^b^
Positive	62 (41.9)		7 (41.2)		
Negative	86 (58.1)		10 (58.8)		
**Gestational age when the maternal PCR was positive**	*n* = 57		*n* = 7		0.4^d^
Median (in weeks)		28 (25–33)		36 (32–39)	
< 14 weeks	2 (3.5)		0		
14 to 28 weeks	24 (42.1)		1 (14.3)		
28 to 41 weeks	31 (54.4)		6 (85.7)		
**Interval between maternal positive PCR and birth**	*n* = 57		*n* = 7		0.7^d^
Median (in days)		78 (36–102)		21 (4–39)	
0 ≥ 91 days	37 (64.9)		6 (85.7)		
91 ≥ 182 days	18 (31.6)		1 (14.3)		
> 182 days	2 (3.5)		0		
**Maternal hospitalization for SARS-CoV-2 infection**					
Yes	3 (1.4)		0		1.0^d^

*Abbreviations*: *IgG* Immunoglobulins G, *IQR* Interquartile range, *OR* Odds ratio, *PCR* Polymerase chain reaction, *UC* Umbilical cord

^a^Wilcoxon-Mann–Whitney test

^b^Chi^2^ test

^c^Kruskal-Wallis test

^d^Fischer’s exact test

In our cohort, only three women were hospitalized for SARS-CoV-2 infection during pregnancy and all of them transferred IgG antibodies to their newborns.

Two women with worsening symptoms in the postpartum period were admitted to ICU with oxygen therapy. In these two cases, the PCR tests were positive eight and three days before delivery and maternal IgG levels were 20 and 15 AU/ml, respectively. Neonatal IgG levels were negative in both cases (Table 4).

The level of IgG in the UC was correlated with the level of IgG in the mother (rho 0.84; *p* < 0.001). There was also a correlation with the cumulative number of maternal pregnancy complications (rho 0.24; *p* < 0.001), with the interval between a positive maternal PCR for SARS-CoV-2 and delivery (rho 0.28; *p* = 0.02) and with the cumulative number of symptoms (rho 0.18; *p* = 0.004).

Mothers who tested positive by PCR less than one week before to delivery had a median IgG level of 6.4 U/mL, with 25% of infants being seronegative. In contrast, among participants who infected more than four weeks prior to birth, the median IgG level at delivery was 20.5 U/mL, and we observed that 96% of infants had detectable IgG in cord blood. Seronegative newborns were observed to have a shorter interval between maternal PCR positivity and delivery than seropositive newborns.

### Placental transfer ratio

**Fig. 2:**
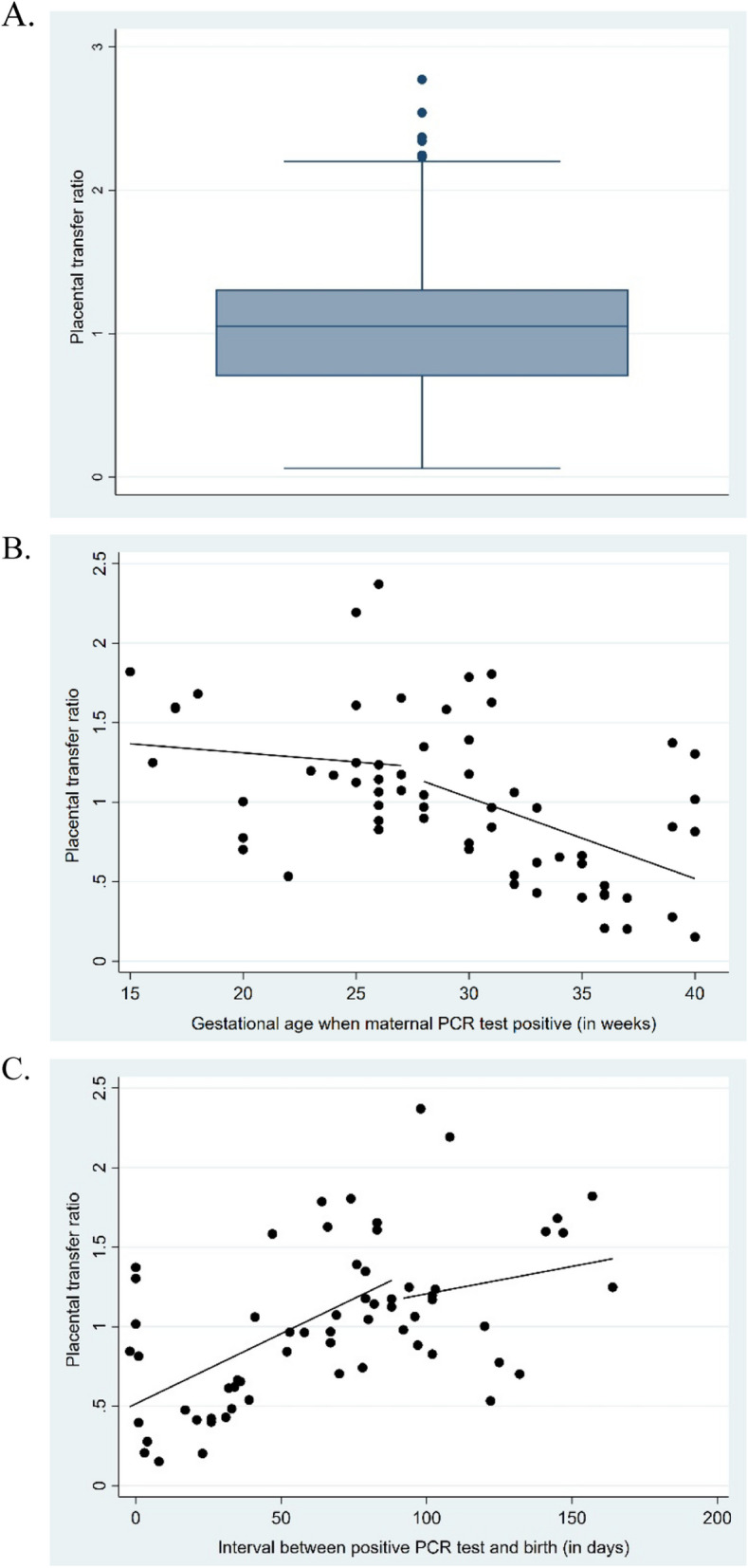
Placental transfer ratio. **a** The box plot showing the placental transfer ratio of all cases. **b** Scatter plot illustrating the relationship between placental transfer ratio and gestational age (in weeks) at which a positive PCR was detected (focusing on the second and third trimesters only). **c** Scatter plot illustrating the relationship between placental transfer ratio and the number of days between birth and positive PCR test result

The median PTR was 1.05, with the highest value of 2.8 (Fig. 2). The longer the delay between a PCR-positive test and delivery, the higher the PTR (*p* < 0.001). Additionally, the PTR increased with younger GA at the time of the PCR-positive test (*p* = 0.02); each additional day increased the PTR by 0.003 (*p* < 0.001).

### Vertical transmission

Of 209 UC samples acquired from 257 mothers who tested positive for IgG, only one newborn tested positive for IgM antibodies (IgM 2.83 AU/mL) against SARS-CoV-2 S1 (0.4%). This newborn IgG level was 71.2 AU/mL. The mother, a primiparous woman in good health, tested negative for IgM (Figure S1). We have not performed confirmatory testing and cannot rule out the possibility of a false positive IgM result due to interference or cross-reactivity.

## Discussion

### Maternal seroprevalence

The seroprevalence of SARS-CoV-2 specific IgG antibodies in our study population was 21.6%. This percentage aligns with the findings of other studies of unvaccinated women carried out in different regions during the initial wave of the coronavirus disease (COVID-19) pandemic [32–34]. Maternal seropositivity was associated with grand multiparity and maternal origin from Northern or Sub-Saharan Africa. The elevated risk observed in grand multiparous women may be attributed to their increased exposure to potential sources of infection. After adjusting for confounding factors, we found that North African origin was associated with a higher risk of maternal seropositivity. A meta-analysis including 18,728,893 participants, suggests that ethnicity may contribute to the likelihood of contracting SARS-CoV-2 infection [35]. Although, some other possible confounders, e.g. large households, respect or not of preventive measures were not investigated.

### Placental transfer of IgG

Our CoVerT cohort showed significant placental transfer of antibodies (81%), consistent with findings from smaller cohort studies [6, 7, 15, 23, 25, 26]. Newborns testing positive for antibodies at umbilical cord were linked with elevated maternal IgG levels, a longer interval between infection and delivery, maternal infection occurred during the early stages of the second and third trimesters of pregnancy, and an accumulation of maternal COVID-19 symptoms [9, 10, 36, 37].

In our cohort, most women were asymptomatic, with imprecise timing of infection. Whenever timing could be estimated, seronegative newborns were born to mothers with a positive PCR test later in pregnancy compared to those who were seropositive. When pregnant women contract COVID-19 towards the end of the pregnancy, maternal IgG antibodies may still be rising, resulting in potentially lower IgG transfer to the fetus [22, 36, 38, 39]. The absence of antibodies observed in newborns of seropositive mothers who contracted infection in the last four weeks of pregnancy, may be attributed to the possible alteration of the Fc glycosylation of SARS-CoV-2 IgG antibodies, influenced by early inflammatory responses [21, 23, 26, 27, 40, 41]. It has been suggested that this modification may normalize gradually over time [22, 26, 27, 37], thereby enabling the typical placenta antibody transfer when there is a sufficient interval between infection and delivery.

In our cohort, the median PTR was calculated at 1.05. A higher PTR was consistently observed when maternal SARS-CoV-2 infection occurred earlier in the second or third trimester, particularly when there was a longer interval between the positive PCR test and delivery, but still within the second and third trimesters. These findings align with previous studies [8, 37, 40] that have reported similar trends in PTR variation concerning the timing of infection and delivery [22, 42, 43]. Due to limitations in our data, we were unable to analyze PTR values for infections occurring in the first trimester.

By understanding the dynamics of antibody transfer and their implications for fetal health following a natural SARS-CoV-2 infection, we can improve vaccination strategies to optimize protection for neonates.

### Strengths and limitations

Our study has some limitations. The timing of SARS-CoV-2 infection, and subsequent antibody production onset, remains unknown. We conducted a retrospective review of medical records, which limited our ability to assess COVID-19 symptoms during pregnancy.

Although comparable between the two groups of women, complication rates were low in both groups: caesarean section (8.7%) and prematurity (1.3%). Participation in the trial had not been systematically offered to women presenting with preterm labor or with a planned cesarean section.

We did not perform a PCR-based diagnostic test for SARS-CoV-2 on all newborns at birth, as this test was considered too invasive to be performed routinely. Furthermore, the clinical interpretation of results in newborns is not always straightforward, due to maternal contamination [44].

Our data from a cohort of unvaccinated pregnant women more than two years ago may be of less clinical value now, but our CoVerT cohort remains the largest group of pregnant women and their newborns who have undergone SARS-CoV-2 antibody testing at birth. Worldwide, not all women who are pregnant or hoping to become pregnant have access to vaccination.

The extensive data on sociodemographic and clinical outcomes for pregnant women and neonates is a solid advantage of this study. In addition, none of the women in our study were vaccinated, which allowed the analysis of antibodies induced solely by natural infection.

## Conclusion

In our study of a cohort of unvaccinated pregnant women who gave birth in Belgian maternities after 35 weeks of pregnancy during the initial year of the COVID-19 pandemic, we found that about one in five women had detectable anti-SARS-CoV-2 spike protein IgG antibodies at the time of delivery. We showed that transplacental transfer of SARS-CoV-2 IgG was significant, particularly when the maternal infection was more severe, occurred earlier in 2nd or 3rd trimester of pregnancy or resulted in higher IgG titers. Only one newborn was found to have IgM in cord blood. These findings provide evidence that maternal anti-SARS-CoV-2 IgG molecules are transferred to the fetus. This underscores the importance of vaccination during pregnancy as a potential strategy to protect newborns against SARS-CoV-2 infection.

### Supplementary Information

Supplementary Material 1.

## Data Availability

Data is provided within the manuscript or supplementary information files. Data cannot be shared publicly because of confidentiality issues and potential identifiability of sensitive data as identified within the Research Ethics Committee application / approval. Requests to access the data can be made by contacting an.vercoutere@hubruxelles.be.

## Abbreviations

SARS-CoV-2: Severe acute respiratory syndrome coronavirus 2
IgG: Immunoglobulin type G
IgA: Immunoglobulin type A
IgM: Immunoglobulin type M
S: Spike protein
N: Nucleocapsid (protein & RNA genome)
GA: Gestational age
IgG1: Immunoglobulin type G subtype 1
IgG2: Immunoglobulin type G subtype 2
UC: Umbilical cord
CoVerT: Covid VERtical Transmission
REDCap: Research Electronic Data Capture
PCR: Polymerase chain reaction
ICU: Intensive care unit
sd: Standard deviation
OR: Odds ratio
aOR: Adjusted odds ratio
COVID-19: Coronavirus disease 2019
