# Supplementary material for: Seroprevalence and placental transfer of SARS-CoV-2 antibodies in unvaccinated pregnant women
Source: BMC Infect Dis. 2024 May 21;24:509. doi: 10.1186/s12879-024-09399-6 (PMC11110414; doi:10.1186/s12879-024-09399-6)

**Supplementary files**

**Tables S1** Variables associated with SARS-CoV-2 infection. Comparison between participants with and without SARS-CoV-2 spike protein IgG antibodies

**Table S2** Logistic analysis of seroprevalence (with and without availability of SARS-CoV-2 PCR test)

**Table S3** Comparison of sociodemographic and gestational characteristics according to whether there is placental transfer of IgG (only maternal IgG positive antibodies are considered).

**Table S4** Comparison of neonatal outcome according to whether there is placental transfer of IgG (only maternal IgG positive antibodies are considered)

**Figure S1** Timeline of the pregnancy with detection of IgM antibodies in the umbilical cord blood of the newborn.

**Figure S2** Confidence ellipse expressing the association between neonatal IgG and maternal IgG when the latter is positive.

**Table S1** Variables associated with SARS-CoV-2 infection. Comparison between participants with and without SARS-CoV-2 spike protein IgG antibodies.

| **SARS-CoV-2 related variables** | **ALL**  **n (%)** | **Maternal IgG positive** | **Maternal IgG negative** | **p value / OR** |
| --- | --- | --- | --- | --- |
| **Interval PCR test– delivery (days)**  Median (IQR)  Min, max | 612  0 (0-2)  -5, 267 | 163  1 (0-53)  -2, 249 | 449  0 (0-1)  -5, 267 | **p < 0.001 °** |
| **PCR test**  Positive  Negative | 617  81 (13.1)  536 (86.9) | 166  67 (40.4)  99 (59.6) | 454  14 (3.1)  437 (96.9) | **p < 0.001** *  **OR 21.1** CI[11.41-39.11]  ref |
| **Symptoms related to COVID**  Yes  No  Fever  Dyspnoea | 1192  56 (4.7)  1137 (95.3)  14  10 | 258  41 (15.9)  217 (84.1)  10  5 | 935  15 (1.6)  920 (98.4)  4  5 | **p < 0.001 ***  **OR 11.6** CI[6.30-21.32]  ref  **OR 9.4** CI[2.93-30.31]  **OR 3.7** CI[1.06-12.85] |
| **Cumulated symptoms**  0  1  > 1 | 1137 (95.3)  24 (2.0)  32 (2.7) | 217 (84.1)  19 (7.4)  22 (8.5) | 920 (98.4)  5 (0.5)  10 (1.1) | **p < 0.001** *  Ref  **OR 16.1**  **OR 9.3** |

* Chi square test

° Mann-Whitney-test

Abbreviations PCR = polymerase chain reaction

IQR = inter quartile range

OR = odds ratio

**Table S2**  Multivariable analysis of seroprevalence

1. Table with availability PCR test


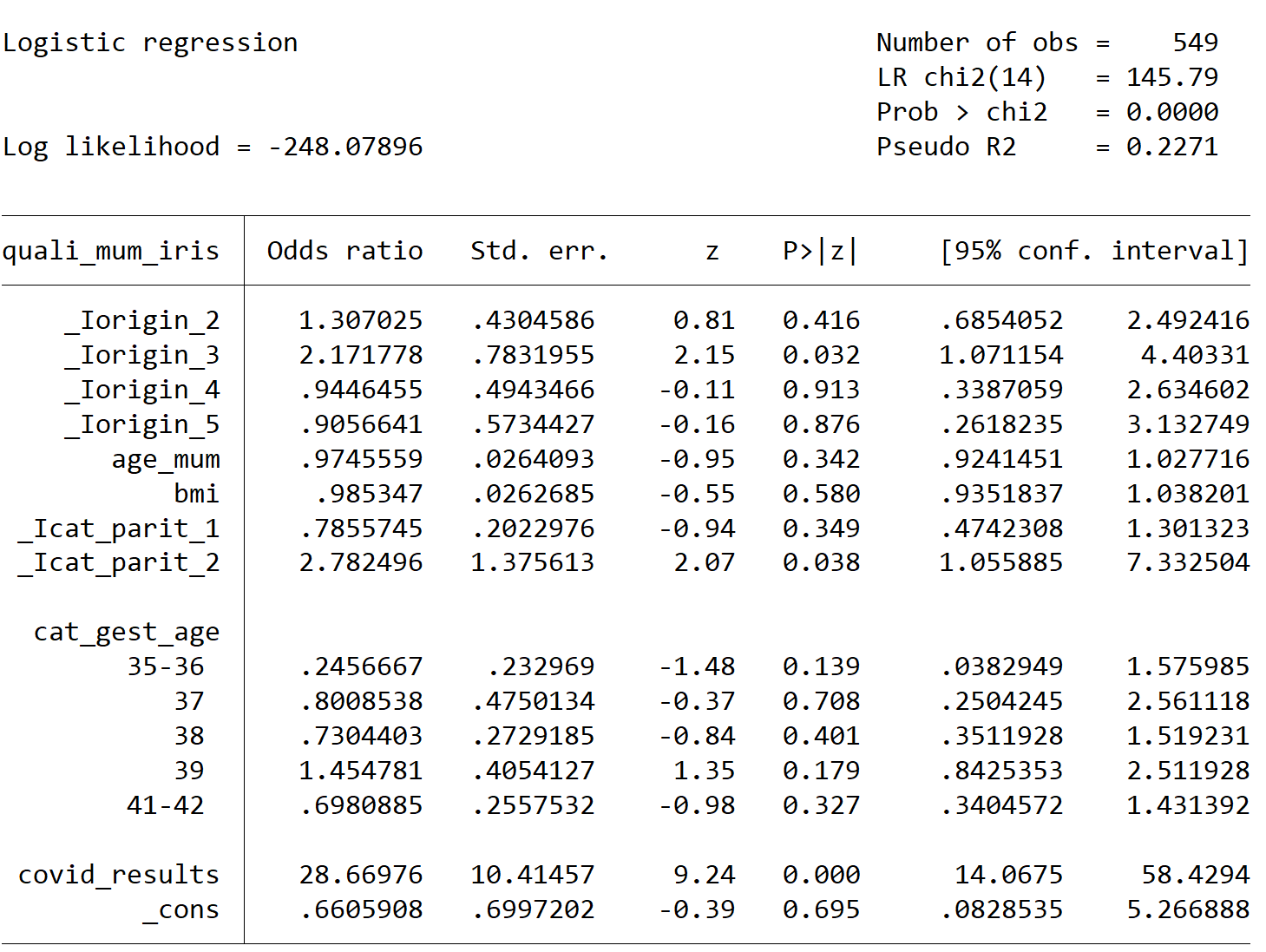


1. Table without availability PCR test


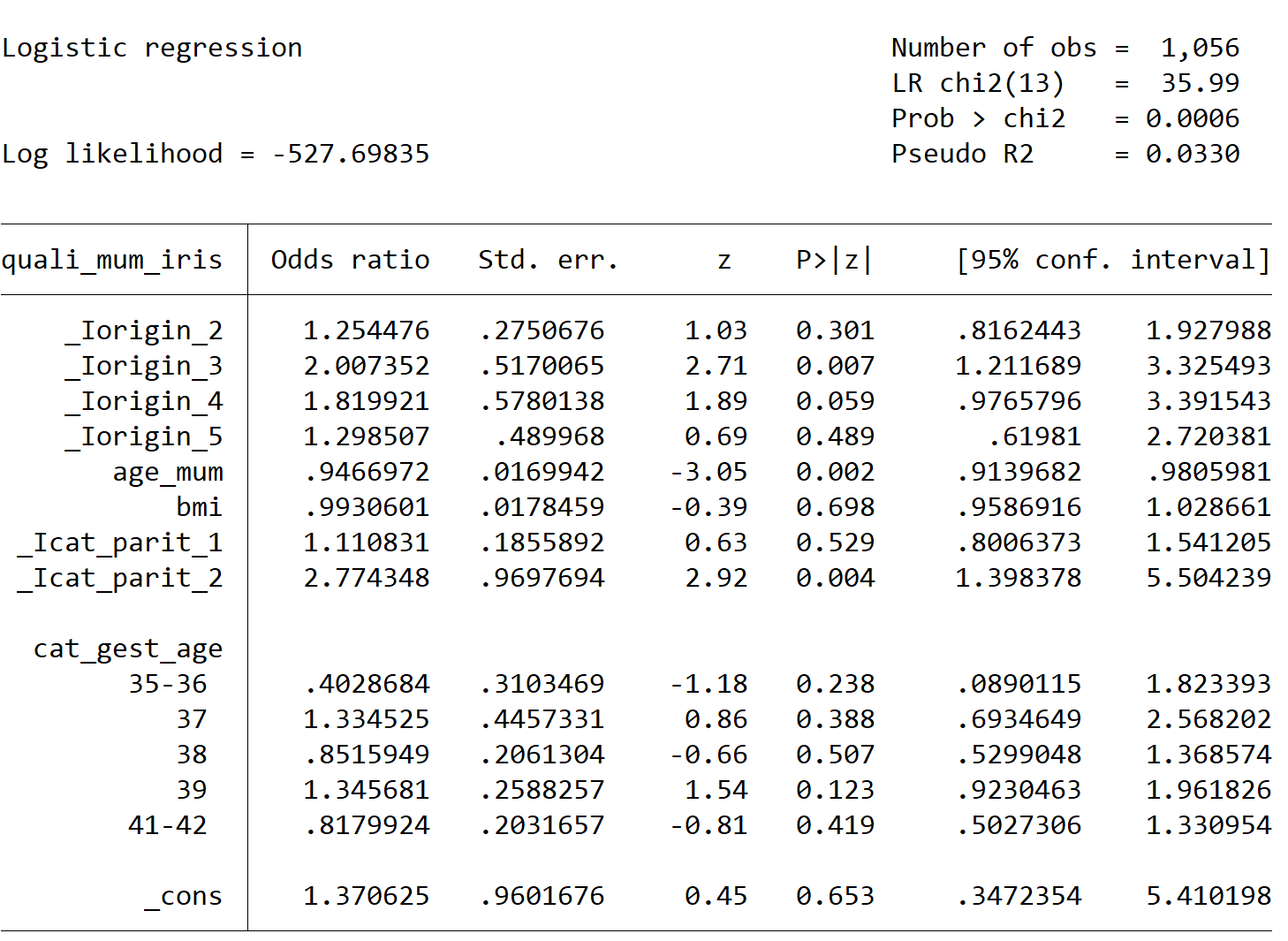


**Table S3** Comparison of sociodemographic and gestational characteristics according to whether there is placental transfer of IgG (only maternal IgG positive antibodies are considered).

| **Socio-demographic and pregnancy characteristics** | **IgG UC**  **positive** | | | **IgG UC**  **negative** | | | **p value** | |
| --- | --- | --- | --- | --- | --- | --- | --- | --- |
|  | **N (%)** | **Mean (sd)* / Median (IQR)** | **N (%)** | | **Mean (sd)* / Median (IQR)** |  | |  |
| **Maternal age** (years) | 209 | 31.7 (4.73) * | 48 | | 32.4 (4.5) * | 0.30 ^a^ | |  |
| **Smoking**  Yes  No | 7 (3.4)  201 (96.6) |  | 4 (8.7)  42 (91.3) | |  | 0.12ᵇ | |  |
| **BMI (kg/cm²)**  < 20  Normal (20-25)  Overweight (25-30)  Obesity (>= 30) | 185  37 (20.0)  95 (51.4)  31 (16.8)  22 (11.9) | 23.7 (4.3) * | 39  4 (10.3)  24 (61.5)  11 (28.2)  5 (12.8) | | 23.5 (3.6) * | 0.82 ^a^ | |  |
| **Parity**  Nulliparity  1-2  Grande multiparity (>= 3) | 209  106 (50.7)  86 (41.1)  17 (8.1) | 0 (0-1.0) | 48  16 (33.3)  28 (58.3)  4 (8.3) | | 1.0 (0-1.0) | 0.053 ^c^ | |  |
| **Intake medication during pregnancy**  Yes  No | 53 (25.4)  156 (74.6) |  | 18 (37.5)  30 (62.5) | |  | 0.09 ^e^ | |  |
| **Pregnancy complications**  Yes | 93 (44.5) |  | 29 (60.4) | |  | 0.06^b^ | |  |
| **Type of birth**  Vaginal birth  Instrumental birth  Primary C-section  Secondary C-section | 168 (80.8)  26 (12.5)  2 (1.0)  12 (5.8) |  | 37 (78.7)  7 (14.9)  1 (2.1)  2 (4.3) | |  | 0.70 ^d^ | |  |
| **Gestational age at birth**  36 - 37 weeks  37 < 38 weeks  38 < 39 weeks  39 < 40 weeks  40 < 41 weeks  > 41 weeks | 209  2 (1.0)  18 (8.6)  30 (14.4)  67 (32.1)  60 (28.7)  32 (15.3) | 39.0  (39.0 – 40.0) | 48  0  3 (6.3)  10 (20.8)  14 (29.2)  16 (33.3)  5 (10.4) | | 39.0  (38.0 – 40.0) | 0.74 ^c^ | |  |

* Presentation of mean (normal distribution of variables)

^a^ test Mann-Whitney

^b^ test Fischer

^c^  test Kruskal-Wallis

^d^ test Chi²

Abbreviations: sd = standard deviation; IQR = interquartile range ; BMI = body mass index (kg/cm²);

**Table S4** Comparison of neonatal outcome according to whether there is placental transfer of IgG (only maternal IgG positive antibodies are considered).

| **Neonatal outcome** | **IgG UC**  **positive** | | | **IgG UC**  **negative** | | | **p value** | |
| --- | --- | --- | --- | --- | --- | --- | --- | --- |
|  | **N (%)** | **Mean (sd)* / Median (IQR)** | **N (%)** | | **Mean (sd)* / Median (IQR)** |  | |  |
| **Birth weight** |  | 3371.3 (443.7)* |  | | 3387.9 (403.2)* | 0.81 ^ | |  |
| **Apgar at 5 min**  Apgar 5 min < 7  Apgar 5 min >= 7 | 2 (1.0)  207 (99.0) |  | 1 (2.1)  47 (97.9) | |  | 0.46 ° | |  |
| **pH**  pH >= 7.3  pH < 7.3 | 85 (40.7)  124 (59.3) |  | 19 (39.6)  29 (60.4) | |  | 0.90 * | |  |
| **NICU admission**  Yes | 13 (6.2) |  | 4 (8.3) | |  | 0.53 ° | |  |
| **Breastfeeding**  Yes, exclusively  Yes, but mixed  No, artificial | 176 (84.6)  15 (7.2)  17 (8.2) |  | 37 (78.7)  2 (4.3)  8 (17.0) | |  | 0.18 ° | |  |
| **Neonatal demise**  Yes | 0 |  | 1 (2.1) | |  | 0.81 ° | |  |
| **Respiratory support**  None  High flow  Nasal CPAP  Mechanical ventilation | 191 (91.4)  1 (0.9)  16 (7.7)  0 |  | 44 (91.7)  0  2 (4.2)  2 (4.2) | |  | 0.091 ° | |  |
| **COVID-19 PCR test**  *(3 missings)*  Yes, done  No, not done | 52 (25.2)  154 (74.8) |  | 9 (18.7)  39 (81.3) | |  | **0.02 °** | |  |
| **Result PCR test**  Negative  Positive | 16 (30.8)  36 (69.2) |  | 7 (77.8)  2 (22.2) | |  | **0.02 °** | |  |

° Fisher exact test

* Chi² test

^ t-test

Abbreviation CPAP = continuous positive airway pressure; IQR = inter quartile range; PCR = polymerase chain reaction test;

sd = standard deviation;

**Figure S1** Timeline of the pregnancy with detection of IgM antibodies in the umbilical cord blood of the newborn.

Abbreviation: PCR = polymerase chain reaction. IgG = antibodies of type G. HC = head circumference.
w = weeks. d = days


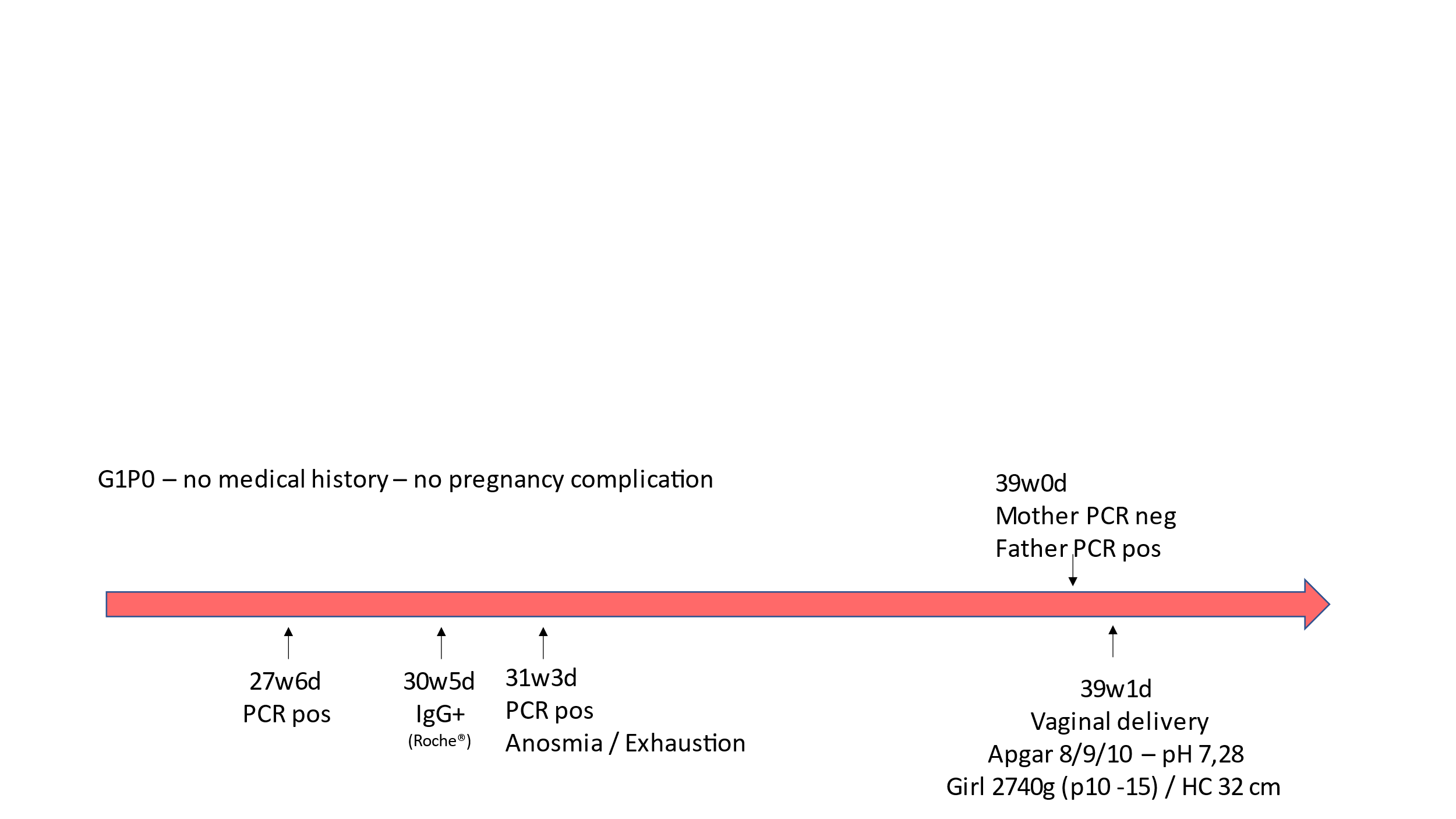


During the second trimester, her partner had contracted a SARS-CoV-2 infection. At 27 weeks and 6 days of gestation the mother tested positive by PCR. At 31 weeks she developed anosmia and fatigue, and another positive PCR test confirmed SARS-CoV-2 infection. At 30 weeks 5 days of gestation she underwent a qualitative IgG antibody test (Roche®) in another laboratory which again was positive.

On admission to the hospital at 39 weeks’ gestation, the mother had a negative PCR test. She subsequently gave birth, vaginally, to a healthy baby girl with a birthweight of 2740g (p10-p15), with good neonatal adaptation and no complications during the early neonatal period.

**Figure S2** Confidence ellipse expressing the association between neonatal IgG and maternal IgG when the latter is positive.


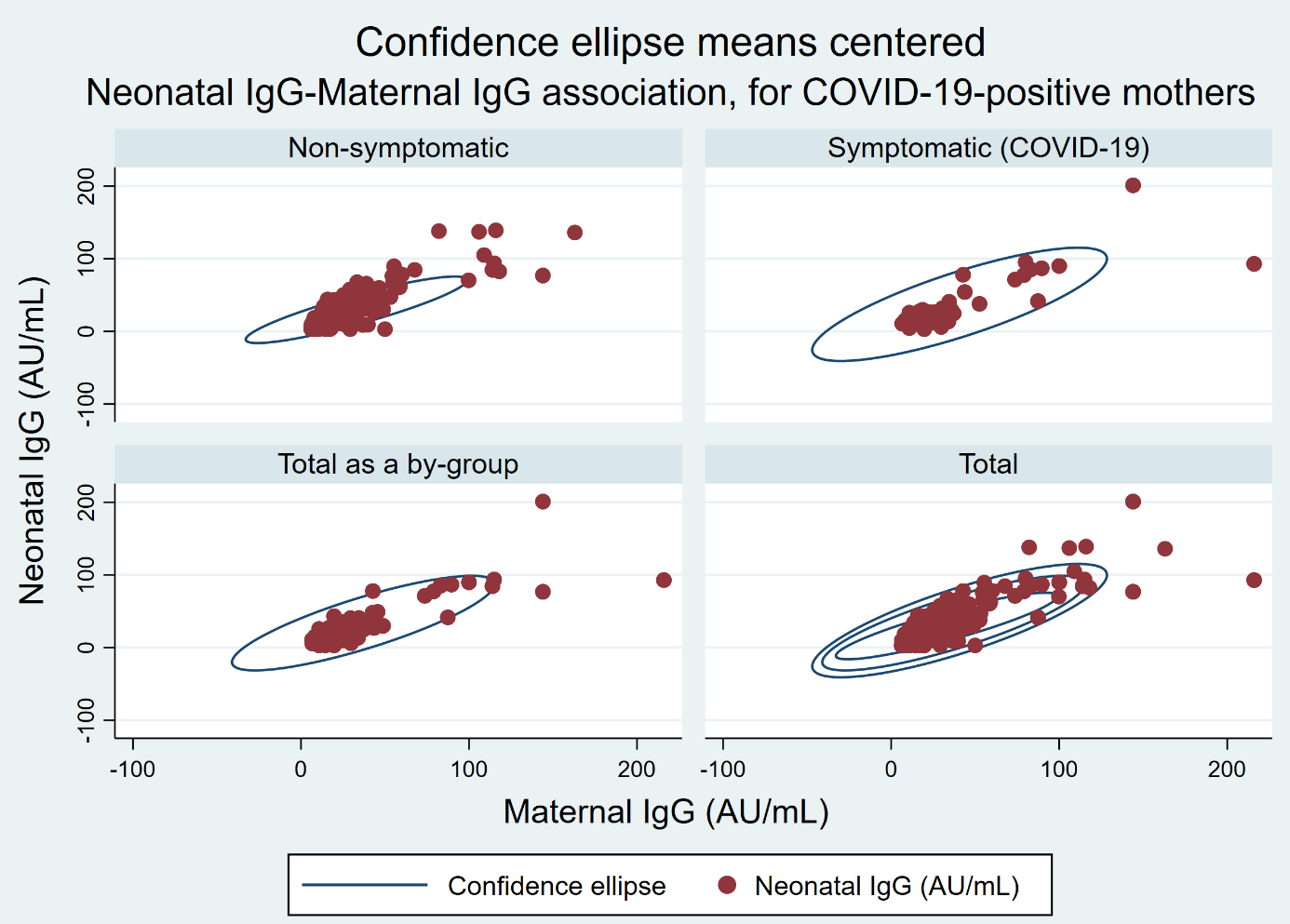

Supplement: Supplementary file 1 — Supplementary Material 1. [file 12879_2024_9399_MOESM1_ESM.docx]
